# Shifts in Demography in Changing Ecological Conditions in a Dependent‐Lineage Population of Harvester Ant Colonies

**DOI:** 10.1002/ece3.74228

**Published:** 2026-08-23

**Authors:** Felix Glinka, Erik B. Steiner, Eyal Privman, Deborah M. Gordon

**Affiliations:** ^1^ Department of Evolutionary and Environmental Biology, Institute of Evolution University of Haifa Haifa Israel; ^2^ InfoGraphics Lab University of Oregon Eugene Oregon USA; ^3^ Department of Biology Stanford University Stanford California USA

**Keywords:** ant colony population, demography, kinship, reproductive senescence

## Abstract

Environmental stress can influence population dynamics and shift selection pressures. We considered the demographic effects since 2011 of intensifying drought in a J1/J2 dependent‐lineage population, monitored since 1988, of the red harvester ant, *Pogonomyrmex barbatus* . A queen must mate with males of both dependent lineages to found a viable colony; mating with the same lineage results in a gyne, while mating with the other lineage results in a worker. We used reduced representation genomic sequencing of workers from 407 colonies, ages 1–3, mostly sampled in 2021–2023, to determine colony lineage, identify sets of colonies with the same mitotype, and estimate kinship between mother–daughter pairs. We found that the rare lineage J1 became even rarer, from 39% in 2011 to 25% in 2023. This creates pressure on J1 to produce more sperm or more males to allow the population to persist. We compared survival and numbers of offspring produced in the two lineages and in sets of colonies with the same mitotypes, using census data. Survival and fecundity were similar among 5 J1 and 6 J2 mitotype groups, showing no evidence for selection favoring any mitotype. We identified 62 mother–daughter colony pairs. These included 14 grandmother‐triplets, with most grandmothers found in J2, suggesting that some families dominate each mitotype group. Within the 62 mother–daughter pairs, those from J1 tended to live longer than J2 colonies, maintaining a low but constant fecundity rate, while J2 colonies showed the highest fecundity at ages 11–17. Our results suggest that increasingly harsh ecological conditions are shaping population dynamics more rapidly than selection for phenotypic traits that could facilitate adaptation.

## Graphical abstract

We compared the demography of two lineages in a dependent lineage population, using data from an annual census in a long term study, and data from the identification of 62 mother–daughter colony pairs. We found differences between the lineages in fecundity and mortality. The lineage ratio has become more skewed toward J2 in the drier period of 2011‐2023 than in 1990‐2010. We examined differences in mitotype groups within each lineage and found no evidence for phenotypic selection.

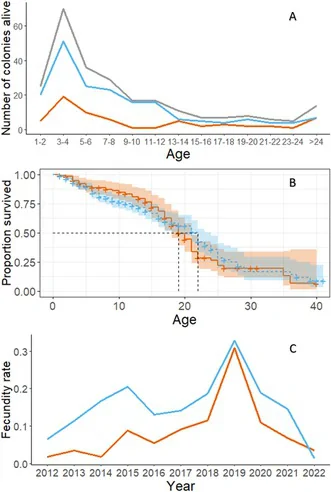

## 1 Introduction

A fundamental question about biotic response to climate change is whether demographic decline will be slow enough to allow for adaptation by natural selection (Bell 2013). One important constraint on the rate of adaptation is generation time, while accelerating climate change is rapidly transforming environmental conditions. Climate change is intensifying arid conditions due to rising temperatures and reduced rainfall in many parts of the world (Hatin et al. 2019). Life history strategies reflect selection pressures created by environmental and physical constraints (Healy et al. 2019), including rising temperatures and water stress (Costa 1995; Holtof et al. 2021; Manlick et al. 2021; Padda et al. 2021; Weaver et al. 2024). There may be a trade‐off between survival and fecundity; reproductive allocation depends on the costs of reproduction and of survival as organisms age (Harris and Uller 2009; Kirkwood and Rose 1991; Lemaître and Gaillard 2017; Stearns 2000).

We draw on a long‐term study of a population of the red harvester ant *Pogonomyrmex barbatus* , censused since 1988 (see photos in figures 2 and 3, Sundaram et al. 2022). Colonies can live for 30 or more years (Gordon and Kulig 1998), founded by a single queen who produces all of the workers, as well as reproductive daughters and sons (Suni et al. 2007). *Pogonomyrmex barbatus* reproduces in an annual mating aggregation of the reproductives from throughout the population of colonies. Newly mated queens found new colonies that live for 20–30 years (Sundaram et al. 2022). Thus, colonies are the reproductive individuals that selection acts upon.

An intensifying drought and decreased rainfall since about 2003 across the southwestern US (Williams et al. 2020) have decreased the food supply for this species. The ants eat the seeds of annual plants and grasses. Regional declines in rainfall (Williams et al. 2020) limit seed production and the availability of seeds at the site. An increasingly limited food supply has led to increased intraspecific competition for foraging area, so that crowded colonies and founding colonies are less likely to survive in years of low rainfall, leading to an overall decrease in population size (Sundaram et al. 2022, figure 1).

We considered the effects of changing ecological conditions on the population dynamics of the unusual dependent lineage system in *P. barbatus* and the closely related species *P. rugosus* (Helms Cahan and Keller 2003; Volny and Gordon 2002; see diagram in Gordon and Friedman 2017, figure 1). As in all Hymenoptera, males are haploid, and females are diploid. The diploid daughters of matings between a female and a male of different lineages are workers; the diploid daughters of matings between a female and a male of the same lineage are reproductives. Each queen must mate with males of both lineages to produce a viable colony. Queens mate with multiple males, with an effective mating frequency with the other lineage of about four (Volny and Gordon 2002).

A previous study of this population of *P. barbatus* through 2011 showed no ecological advantage for either lineage; the lineages did not differ in their tendency to relocate nests, survival, foraging activity, nest mound size, or gyne production (Gordon et al. 2013). However, the lineage ratio tends to be skewed toward J2, as in many dependent‐lineage populations of *P. barbatus* (Anderson et al. 2009; Gordon et al. 2013; Schwander et al. 2007).

**FIGURE 1:**
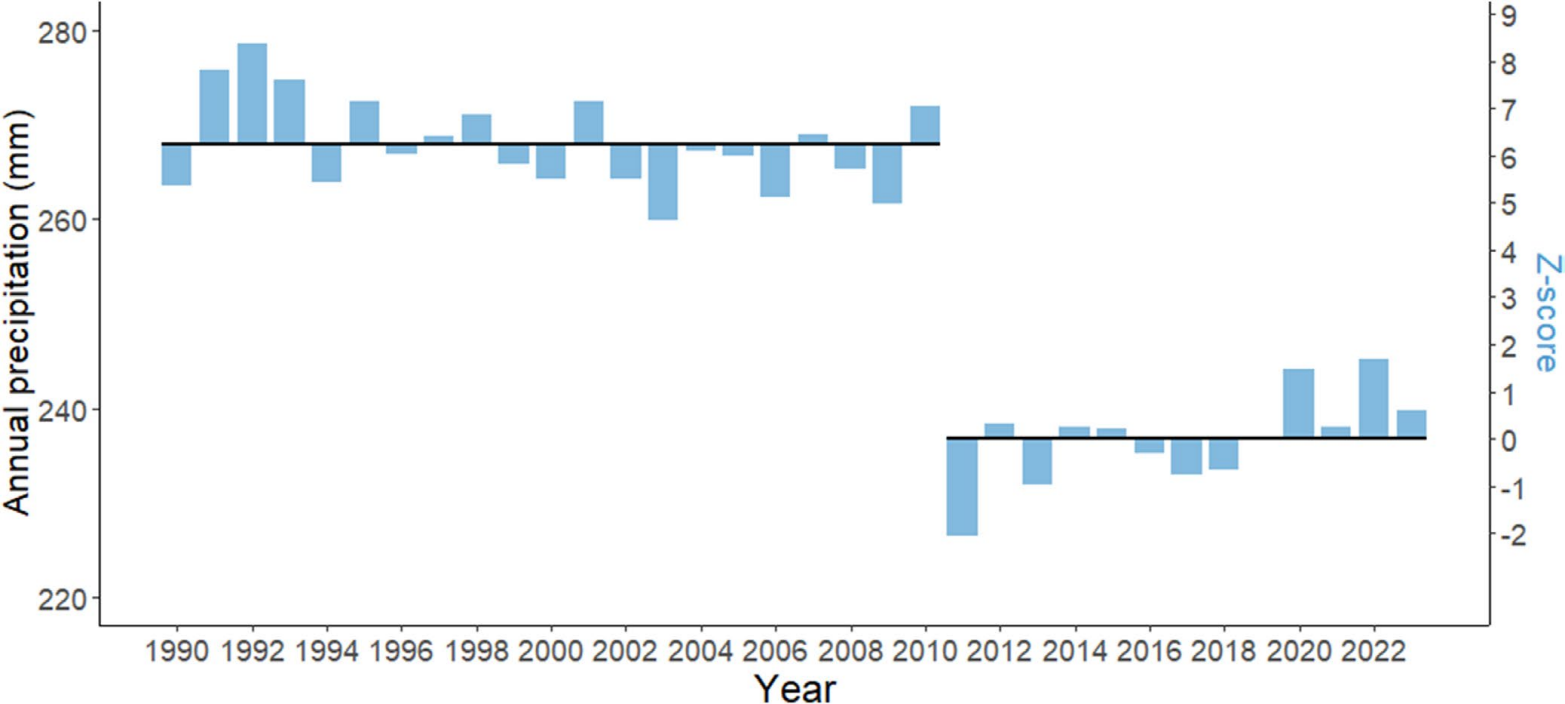
Annual rainfall and *z*‐score for the years 1990–2010 and 2011–2023. Black lines show the means for each period. Blue bars show *z*‐scores relative to each mean.

We investigated changes in the demography of the two lineages in the drier period of 2011–2023, compared to results from 1990 to 2010 (Gordon et al. 2013; Ingram et al. 2013). The study site is situated in a valley about 160 km wide. The food supply for the ants at the site is mostly seeds distributed by the wind (Gordon 1993), and the distance from which seeds are distributed is not known. Rainfall data from a weather station about 65 km from the site show that precipitation has declined from a mean of 267.93 mm 1990–2010 to a mean of 236.75 mm during the period of 2011–2023 considered in this study(Figure 1).

We first considered changes in the lineage ratio since 2011. We then asked whether the lineages differ in the extent to which certain families dominate the population. J1 has 5 mitochondrial haplotypes (mitotypes), while J2 has 6 mitotypes. Because the mitotype is maternally inherited, mother–daughter pairs must belong to the same mitotype group. If strong selection is favoring some heritable phenotypic trait that varies among mitotypes, then some mitotype groups would show higher reproductive success, either in higher fecundity or lower mortality than others. For example, mitotype groups reflect natural selection in fish (Bernatchez 2001; Brunner et al. 2001) and elephants (Eggert et al. 2008). In ants ( *Formica rufa* group, *Formica exsecta* ), divergent mitotype groups in different locations indicate evolutionary pressures that may have led to recent speciation (Goropashnaya et al. 2004, 2007). By contrast, similar survival and production of offspring colonies among mitotype groups in the harvester ant population would suggest that ecological conditions affecting all phenotypes are currently shaping population dynamics.

To compare fecundity and life history in the two lineages, we identified mother–daughter pairs of colonies. We conducted reduced representation genomic sequencing of at least two worker samples from each of 407 colonies, ages 1–3, mostly sampled in 2021–2023. We used these data to find single‐nucleotide polymorphism (SNP) loci and compile a genome‐wide map of genetic polymorphism (Flanagan and Jones 2019). The unusual population genetics of the dependent lineage system challenges standard methods. To address these challenges, we developed a kinship inference method appropriate for the dependent‐lineage system, using lineage‐specific alleles to identify mother–daughter relationships. We examined differences between the two lineages in fecundity and the age of reproduction. In most animals, there is a tradeoff between life expectancy and reproduction (Blacher et al. 2017; Vergara‐Martínez et al. 2024), but there is no evidence for reproductive senescence in this population (Ingram et al. 2013).

## 2 Methods

### 2.1 Census

Since 1988, more than 1200 colonies have been monitored at a long‐term study site near Rodeo, New Mexico, USA, so the ages and locations of all colonies are known (Sundaram et al. 2022). Our genetic analysis of lineage, mitotype group, and kinship used samples of workers from 407 colonies of known age, which were founded between 1983 and 2022, collected at the study site beginning in 2000. We analyzed colony fecundity from 2012 to 2023 and colony mortality from 2012 to 2024. Rainfall data are from a weather station operated by the Western Regional Climate Center in San Simon, AZ, about 65 km from the study site (https://wrcc.dri.edu/WRCCWrappers.py?sodxtrmts+027560+por+por+pcpn+none+msum+5+01+F; taken 27th July 2026).

**FIGURE 2:**
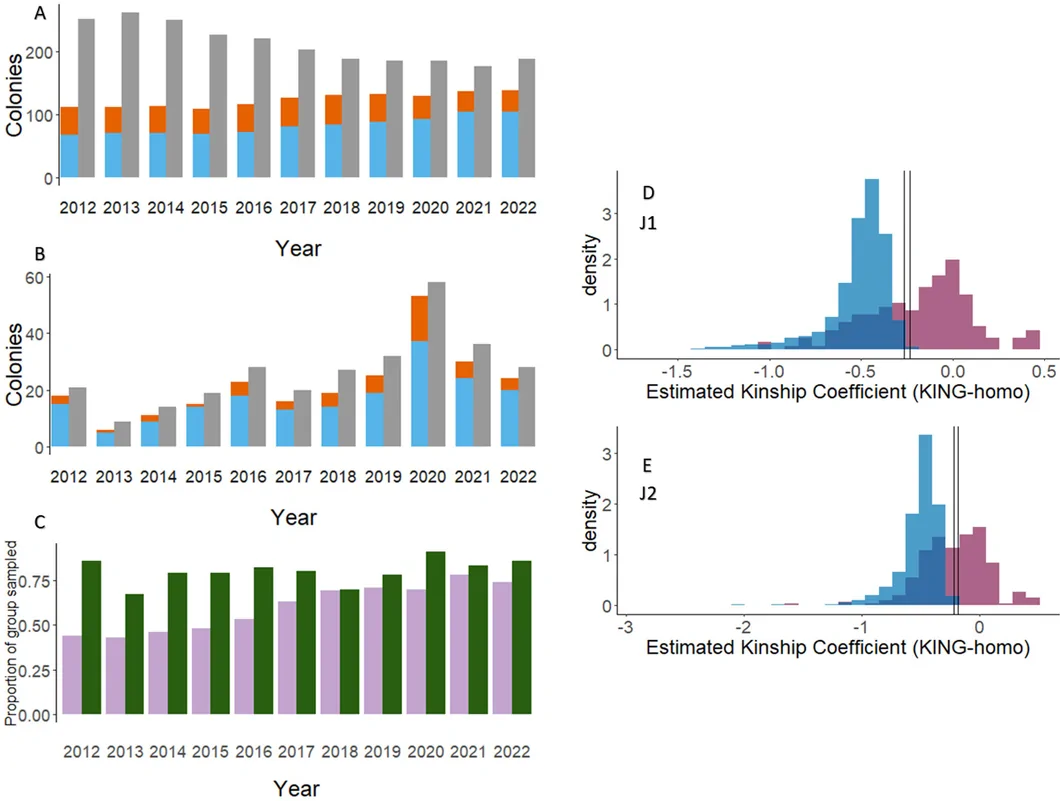
Proportions sampled and kinship coefficients. (A) Number of potential parent colonies in sample by lineage. J1, Orange; J2, Blue and total number of potential parent colonies (gray) per year. (B) Number of 1‐year‐old colonies by lineage in the sample and total number of offspring colonies. J1, Orange; J2, Blue; Total, Gray. (C) Proportion sampled of total potential parent colonies (purple) and of offspring (green). (D, E): Application of the kinship inference method with nestmates as positive controls. The vertical lines show the two thresholds for deciding which colonies are related. The thresholds were determined by the true positive rate (TPR) and false positive rate (FPR) of the nestmates. The purple distribution shows the estimated kinship coefficient for nestmates, and the blue distribution shows the estimated kinship coefficient for non‐nestmates (D: J1, E: J2).

Colonies usually begin to reproduce at age five, with a few colonies that reproduce at age four (Gordon 1995). Here, we assumed that a colony could be a mother at the age of four. Our sample included at least 40% of the potential parent colonies in the population in every year between 2012 and 2022 (Figure 2A,C), and 70% of the offspring colonies, counted as those found at the age of one year (Figure 2B,C). The oldest colony sampled (in 2020) was 36 years old at the time. A higher proportion of the population was sampled in 2018–2021 than in earlier years (Figure 2C).

### 2.2 Mitochondrial Sequencing and Lineage Identification

Universal insect *cox*1 primers modified for *P. barbatus* (forward C1‐j‐1751 (Pb) C1‐N‐2191 (Pb) 5′) were used to amplify a 433 bp portion of the mitochondrial gene *cox*1 for all 1101 worker samples, as in Cahan and Keller (2003). All reactions were run in a 25 μL volume using Q5 Hot Start Polymerase NEB with the following PCR conditions: 98°C for 30 s, followed by 35 cycles of 98°C for 10 s, 56°C for 30 s, and 72°C for 30 s, paused and completed by 72°C for 2 min. PCR products were cleaned using Ampure XP magnetic beads and sent off for Sanger sequencing (Hy Laboratories Ltd). Resulting mitotypes were used to distinguish between the two lineages.

Mitotypes were aligned with *MAFFT* (Yamada et al. 2016) and viewed in *JalView* (Waterhouse et al. 2009). All sequences were trimmed, so each sequence was of comparable size and had no low‐PHRED quality base pairs at its edges. After that, the maximum likelihood gene tree was built using *RaxML* v.8 using the GTRGAMMA substitution model (Stamatakis 2014), which resulted in two clearly distinct monophyletic clades. The lineages were then labeled as J1 and J2 based on previously defined J1 or J2 sequences in each of the clades.

### 2.3 ddRAD Sequencing

Double‐digest Restriction site‐Associated DNA sequencing (ddRAD‐seq) was conducted for 1101 workers from 407 colonies (2–4 workers per colony), using a modified protocol, according to Brelsford et al. (2016), which was based on Parchman et al. (2012). Briefly, genomic DNA was extracted from the head and thorax of individual worker samples using the QIAGEN DNeasy protocol. DNA was digested using the restriction enzymes EcoRI and BfaI, which were chosen for high sequencing efficiency, as estimated using *ddgRADer* (Lajmi et al. 2023). Adaptors containing unique barcodes were ligated to each sample for multiplexing. Ligated products were PCR amplified in replicates of four using Q5 Hot Start Polymerase NEB for 20 cycles with a starting DNA volume of 6 μL. Samples were pooled and size‐selected for fragments of a size of 300–700 bp using BluePippin (Sage Science). The libraries were sequenced on an Illumina HiSeq X sequencer, using a paired‐end, 150 bp read protocol. On average, 1,519,508 reads per sample were obtained.

### 2.4 ddRAD Sequence Data Processing

The resulting reads were quality‐checked with *fastQC* v. 0.12.1 (Andrews 2017). Reads were demultiplexed using the *process_radtags* of the *STACKS2* (v. 2.66) pipeline (Catchen et al. 2011) by their barcodes and then trimmed using the program *PEAR* (Zhang et al. 2014). The resulting reads were mapped to the *P. barbatus* reference genome (J1 genome, Glinka et al. 2026) using *bowtie2* (v. 2.5.3; using the –end‐to‐end and –very‐sensitive parameters) (Langmead and Salzberg 2012). Then the *STACKS2* pipeline (Catchen et al. 2011) was applied to identify SNPs and call genotypes of each sample at each locus. First, ref_map.pl. from *STACKS2* was used to call SNPs, and the *populations* program was used to convert the calls into readable VCF files (using the ‐‐vcf parameter), one VCF file containing SNPs and another containing read haplotypes (where a locus corresponds to the concatenation of all SNPs within the same pair of RAD‐seq reads). The read haplotype VCF has an allelic richness of 5.8 Alleles per locus, allowing for more unique alleles for each lineage. Custom scripts (https://doi.org/10.5281/zenodo.17459734) were predominantly used to process further VCF files. The SNP VCF file was used to add the depths to the respective variants of the read‐haplotype VCF. We used *VCFtools* v. 0.1.13 (Danecek et al. 2011) to filter the resulting read‐haplotype VCF file. We first filtered samples for missing data of over 30% (‐‐missing‐indv, then ‐‐remove), then to limit the number of missing genotypes per locus to 25% (‐‐max‐missing 0.75), requiring at least 1% minor allele frequency (‐‐maf 0.01) per locus, and a minimum depth of 10 reads covering a locus to call the genotype of a sample (‐‐min‐DP 10). In total, we achieved a SNP depth of 76.2823 for every SNP.

### 2.5 Lineage‐Specific Alleles

Because inter‐lineage matings result in sterile workers, there is no gene flow between the two lineages, resulting in an unusual distribution of genotypes in the hybrid workers. This is apparent from the striking level of heterozygosity and the unusual U‐shaped distribution of heterozygosity in workers (Figure S1). We developed a new method to infer relatedness between colonies in a dependent‐lineage population. Workers receive alleles from parents of different lineages. To infer kinship between a pair of mother–daughter colonies using worker samples, we used only the genetic material from the mother's lineage. Workers from a mother–daughter colony pair are expected to share 25% of their maternally inherited genes.

We first identified lineage‐specific alleles using haplotypes consisting of multiple SNPs found within some of the RAD‐tag loci. These are covered by the two 150‐base reads obtained by paired‐end sequencing, which often harbor multiple SNPs. We used a custom Python script (findAllelesInVCF.py) to identify lineage‐specific alleles in the worker genotype data, with additional support from whole‐genome sequencing of 12 haploid male samples from each lineage (10–18× genomic coverage; Glinka et al. 2026). An allele was considered to be lineage‐specific when it was found in at least one male sample of one lineage and not in any male sample of the other lineage, and was in heterozygous genotypes in at least 5% of all the workers, with no more than 1% homozygous genotypes. This may have allowed some alleles that were not completely lineage‐specific, yet were strongly lineage‐biased.

The read‐haplotype VCF file was divided according to the respective lineage‐specific alleles: one VCF file for each lineage, containing only loci with lineage‐specific alleles of the maternal lineage, with samples from colonies that were assigned to this lineage based on the mitotype. We then created a VCF file with the haploid genotypes from the maternal lineage of each sample using another custom script (makeVCFHomozygous.py) that transformed all heterozygous genotypes into homozygous genotypes for the lineage‐specific allele. We left homozygous genotypes unchanged.

### 2.6 Kinship Inference

To identify mother–daughter pairs of colonies, we used lineage‐specific alleles of the queen's lineage to infer kinship between two workers from a mother–daughter colony pair. Since a queen is the daughter of a queen and a father from the same lineage, while a worker is the daughter of a father from the other lineage, a worker from the mother colony and a worker from the daughter colony share genetic material only through the maternal lineage.

We tested for relatedness between all colonies that were potential mother–daughter pairs. As daughter colonies must have the same mitotype as their mothers, mother–daughter colony pairs could occur only within mitotype groups. We tested all colony pairs with an age difference of at least 4 years such that the older one could plausibly be the mother of the younger one. We had 2 workers from most colonies in our sample, so there were 4 relatedness tests between most colony pairs. We used *KING* (Manichaikul et al. 2010) from the *SNPrelate* R package (Zheng et al. 2012, 2017) with the “King‐homo” estimator (for homogeneous populations) to estimate the kinship coefficient between pairs of workers based on the lineage‐specific haploid VCF file.

We calibrated our method using the 490 pairs of nestmates in our sample as positive controls, which are expected to be either full‐ or half‐sisters. We applied the method to all pairs of nestmate and non‐nestmate samples from colonies of each lineage separately. This method produced mostly negative values, even though kinship coefficients should be between 0 and 1. Nevertheless, both lineages showed distinct, although partially overlapping, distributions of kinship coefficient values for nestmates and non‐nestmates (Figure 2D,E). The median kinship coefficients in J1 are −0.147 and −0.472 for nestmates and non‐nestmates, respectively. The two kinship distributions were significantly different for J1 (Kolmogorov–Smirnov test, *D* = 0.65, *p* < 2.2e‐16). Similarly, the median kinship coefficients in J2 are −0.187 and −0.470 for nestmates and non‐nestmates, respectively, and the two kinship distributions were significantly different (Kolmogorov–Smirnov test, *D* = 0.6, *p* < 2.2e‐16). This confirmed that the method detects the statistical signal of relatedness between nestmate pairs relative to the background distribution of non‐nestmate pairs.

We set a first threshold for kinship inference at −0.233 for J1 and −0.179 for J2, based on the kinship distributions of nestmates and non‐nestmates. We then found all pairs of workers from two colonies of the same mitotype group that could be mother–daughter pairs (an age difference of at least 4 years) above the threshold value of the kinship coefficient. We then allowed a less stringent, slightly lower threshold of −0.266 for J1 and −0.219 for J2, for which we require at least two sample pairs from the two colonies to be above the threshold to consider them a mother–daughter colony pair. Because we chose thresholds to minimize false positives, our results do not include all the mother–daughter pairs, as can be seen from the proportion of nestmates below the thresholds in Figure 2D,E. We calculated the true positive rate (TPR) and false positive rate (FPR) per lineage by counting the number of nestmate and non‐nestmate pairs with a kinship coefficient above the threshold. For J1, with the first threshold of −0.233, the TPR is 0.6, and the FPR is 0.003. For J2, with the first threshold of −0.179, the TPR is 0.5, and the FPR is 0.001.

### 2.7 Demographic Changes

#### 2.7.1 Lineage Ratio

We considered how the lineage ratio changed between 2011 and 2023. Using the data from the annual census, we examined differences in survival between the lineages of the 407 colonies in our sample.

#### 2.7.2 Age Distribution

We tested whether living colonies of the two lineages differed in longevity by comparing the distribution of ages of living colonies using the Kolmogorov–Smirnov test.

#### 2.7.3 Survival

We tested whether the lineages differ in survival using the Kaplan–Meier analysis with the *survfit*() function from the survival package (Therneau 2024). We used this test because it is a nonparametric method that makes no assumptions about the survival distribution. We constructed a survival curve for each lineage using all colonies sampled. We then tested whether the survival differed between lineages using the log‐rank test.

#### 2.7.4 Mortality by Year

We tested whether years differed in colony mortality and whether this differed for the two lineages. We used a generalized linear model (GLM) modeling the counts of colony death as the response variable. We chose the final model using its AIC value (Table 1) with the following parameters as fixed effects: (1) year from 2012 to 2024, the years when our sample included more than 40% of all colonies in the population (Figure 2A–C); (2) the ratio of J2 to J1 colonies. We used a Poisson GLM because it is designed for nonnegative integer values and assumes that the variance of the data is a function of the mean, as is typical for count data (Cameron and Trivedi 2012).

**TABLE 1:** Model specification for the GLM to evaluate year‐to‐year and lineage differences in mortality and fecundity. Shown are the model formula, degrees of freedom (df), and band value of the Akaike Information Criterion (AIC). The response variables were the counts of new colonies per year and colonies that died each year.

Model	Formula	df	AIC
*Mortality*			
m0	year*Lineage	4	134.55
m1	year+Lineage	3	132.76
m2	year	2	136.42
*Fecundity*			
m0	year*Lineage	4	178.46
m1	year+Lineage	3	177.67
m2	year	2	256

#### 2.7.5 Fecundity by Year

To test whether years differed in colony fecundity and whether this differed for the two lineages, we used a GLM to model the counts of the number of colonies that were 1‐year‐old as the response variable, and chose the final model based on its AIC value (Table 1). In the final model the fixed effects were: (1) year 2012–2023, the same years included for kinship inference because we had a large enough sample for these years; (2) the lineage ratio J2 to J1. We used a Poisson GLM for the same reasons described above for the mortality analysis.

We asked whether the two lineages differed in the numbers of offspring colonies by calculating the ratio of offspring (1‐year‐old) colonies per potential parent colony. For the offspring colonies of each year, a potential mother colony was a colony of the same lineage that was at least 4 years old, as colonies usually begin to reproduce at age 5, but rarely at age 4 (Gordon 1995; Ingram et al. 2013). The ratio of offspring per potential parent was the number of 1‐year‐old colonies divided by the number of potential mothers that year. We compared the distribution of the ratio of the number of newly founded colonies to the number of potential mother colonies in that year in the two lineages from 2012 to 2022 (Figure 2A–C), using the Kolmogorov–Smirnov test.

#### 2.7.6 Mitotype Groups

To examine the possibility that some heritable phenotypic trait is associated with survival, we compared survival among mitotype groups. Our population sample contained 11 distinct mitotypes, 5 in J1 and 6 in J2. Not all colonies of the same mitotype group are closely related, but all closely related colonies must be in the same mitotype group. We first tested whether the distribution of the number of colonies per mitotype group differs between lineages using the Kolmogorov–Smirnov test. Next, we compared the age distributions of the two largest mitotype groups of each lineage using the Kolmogorov–Smirnov test. Finally, we compared the survival rates of the two largest mitotype groups of each lineage using a log‐rank test to determine if the colonies of the mitotype groups have different patterns in their survival rates.

#### 2.7.7 Mother–Daughter Pairs

We inferred 62 mother–daughter colony pairs. We used these pairs to test for differences in fecundity between lineages and mitotype groups using Kolmogorov–Smirnov tests.

## 3 Results

### 3.1 Lineage Ratio

There were more colonies of J2 than of J1. The proportion of J2 increased from 61% in 2011 to its maximum of 76% in 2022. Of the 407 colonies sampled for this study, 120 were J1, 286 were J2, and 1 colony could not be assigned to a lineage. In some years, J1 produced more offspring than J2 (Figure S2A; GLM *Z* = 3.14, *p* = 0.002; Table 2). The number of J1 colonies in our sample set peaked in 2020, while the number of J2 colonies peaked in 2022 (Table 3).

**TABLE 2:** Model testing year‐to‐year differences between lineages in fecundity. The response variable is the count of new colonies by year per lineage. Shown are the estimates (the calculated regression coefficients), the standard error, the *z* value with its respective *p*‐value.

	Estimate	Std. error	*z*	*p*
(Intercept)	−117.46	37.9	−3.09	0.002
Year colonies were 1 year old	0.06	0.02	3.14	0.002
Lineage Ratio J2/J1	1.26	0.15	8.16	3.41e‐16

**TABLE 3:** Lineage ratio 2011–2023. Shown are the number of colonies of each lineage, the proportion that were J2, and the proportion of colonies of each lineage alive the previous year that had died by the indicated year.

Years	# J1 colonies	# J2 colonies	Proportion of J2 colonies	Proportion J1 colonies died	Proportion J2 colonies died
2011	58	90	60.81	0.08	0
2012	60	103	63.19	0.02	0.02
2013	56	104	65.00	0.08	0.04
2014	57	108	65.45	0.02	0.04
2015	53	116	68.64	0.09	0.05
2016	57	131	69.68	0.02	0.02
2017	58	140	70.70	0.03	0.03
2018	63	151	70.56	0	0.02
2019	63	166	72.48	0.09	0.02
2020	71	193	73.11	0.1	0.05
2021	69	208	75.09	0.1	0.04
2022	68	209	75.45	0.07	0.08
2023	64	189	74.70	0.09	0.1
Total	120	286	70.44	—	—

### 3.2 Age Distribution

**FIGURE 3:**
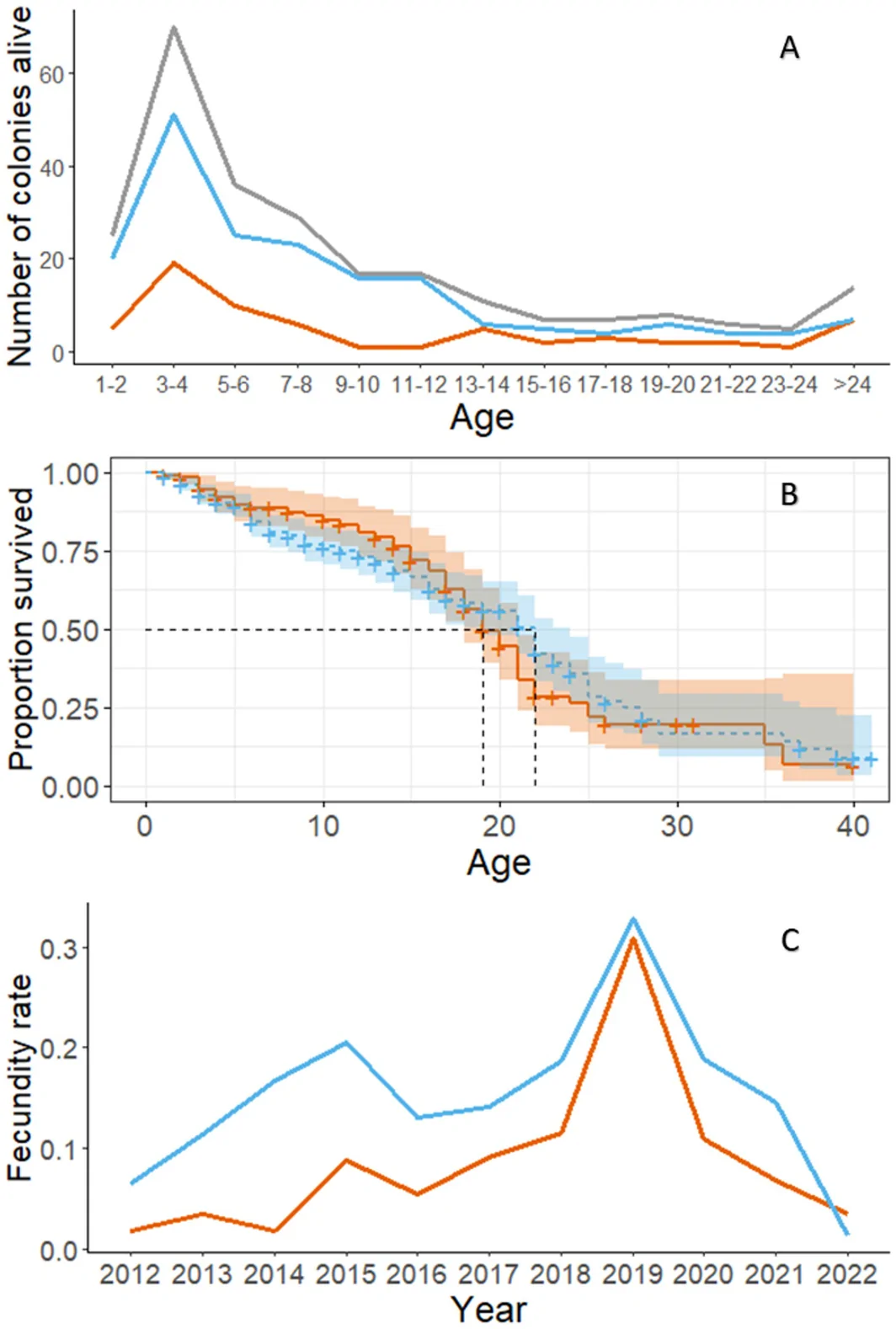
Survival and age distribution. (A) Age distribution in 2023 of the 252 surviving colonies in our sample. (B) Kaplan–Meier survival plot of the two lineages. The dotted line shows the survival probability of 50% for each lineage. J1, Orange; J2, Blue. Number of colonies of age 0: J1 120, J2 286; 10 years.: J1 66, J2 107; 20 years.: J1 29, J2 46; 30 years.: J1 6, J2 7; 40 years.: J1 1, J2 2. Shading shows the 95% confidence interval. J1, Orange; J2, Blue. C: Fecundity rate (potential offspring per possible parent) for each lineage by year. J1, Orange; J2, Blue.

The two lineages differed significantly in age distribution (Kolmogorov–Smirnov test, *D* = 0.26, *p* = 0.01; Figure 3A). J2 had many more colonies younger than 13 years old, while the number of colonies older than 13 years was only slightly higher for J2 than for J1. In both lineages, the peak of the age distribution was ages 3–4 years, but in the J2 lineage, the age distribution was more skewed toward 3–4 years than in J1 (Figure 3A).

### 3.3 Survival

The survival curves of the two lineages were not significantly different (log‐rank test, *χ* ^2^ = 0.1, df = 1, *p* = 0.8, Figure 3B). The median survival time was 19 for J1 and 22 for J2 (Figure 3B). The death rate for J1 was lower than for J2 colonies for ages 1–10, then rose after age 10, and declined after age 24. After age 24, survival rates were similar in both lineages.

### 3.4 Mortality by Year

As of 2024, 155 of the colonies in our sample had died. The highest number of colony deaths occurred from 2022 to 2023 (4 colonies in J1, 20 colonies in J2; Figure S2B). For J2, the year with highest mortality was 2023; for J1, it was 2020 (Table 3). Overall, there was a significant difference between the two lineages in mortality rate from 2012 to 2024 (*Z* = 2.33, *p* = 0.02; Table 4). Mortality also differed across these years (GLM, *Z* = 5.28, *p* = 1.27e‐07; Table 4). Before 2015, there were no visible differences between the lineages in mortality (Figure S2B).

**TABLE 4:** Model testing year‐to‐year differences between lineages in mortality. The response variable is the number of colonies that died each year per lineage. Shown are the estimate (the calculated regression coefficients), the standard error, the *z* value with its respective *p*‐value.

	Estimate	Std. error	*z*	*p*
(Intercept)	−258.12	49.16	−5.25	1.51e‐07
Year colonies died	0.13	0.02	5.28	1.27e‐07
Lineage Ratio J2/J1	0.42	0.18	2.33	0.02

### 3.5 Fecundity by Year

In most years from 2011 to 2022, the majority of new colonies were in the J2 lineage (Figure 2A–C), maintaining the skewed lineage ratio (Figure 3A). The two lineages differed significantly in the number of offspring colonies per year (*Z* = 1.11, *p* = 3.41e‐16; Table 2), and in fecundity rate (potential offspring per possible parent) (Kolmogorov–Smirnov Test, *D* = 0.64, *p* = 0.02; Figure 3C). From 2012 to 2015, the ratio of offspring colonies per potential mother colonies per year was less than 0.2 for both lineages. Fecundity was highest in 2019, with a peak of 0.31 for J1 and 0.33 for J2, and then declined to a value below 0.05 by 2022 (Figure 3C). J2 had a higher fecundity rate than J1 in the years up until 2015, but this gap between the lineages closed in 2019 and 2022 (Figure 3C).

### 3.6 Mitotype Groups

Five mitotype groups were identified from 120 colonies in J1 and six mitotype groups from 286 colonies in J2 (Table 5; sequences in Table S1). For the remaining 26 colonies, the sequences were sufficient to identify the lineage, but not a mitotype group. Both lineages have a similar number of colonies per mitotype group (Kolmogorov–Smirnov test, *D* = 0.27, *p* = 0.93).

**TABLE 5:** Number of colonies and identified mothers per mitotype group per lineage.

Mitotype Grou##p	Number of colonies	Number of identified mothers
*J1*		
J1A	82	17
J1B	12	2
J1C	11	1
J1D	3	0
J1E	2	1
*J2*		
J2A	192	31
J2B	50	8
J2C	9	1
J2D	9	0
J2E	8	1
J2F	2	0

Mitotype groups were labeled according to the number of colonies, with J1A (82 colonies, 75% of all) and J2A (192 colonies, 71% of all) the largest in each lineage, J1B (12 colonies; 11% of all J1 colonies) and J2B (50 colonies; 19% of all J2 colonies) the second largest, and so on (Table 5).

**FIGURE 4:**
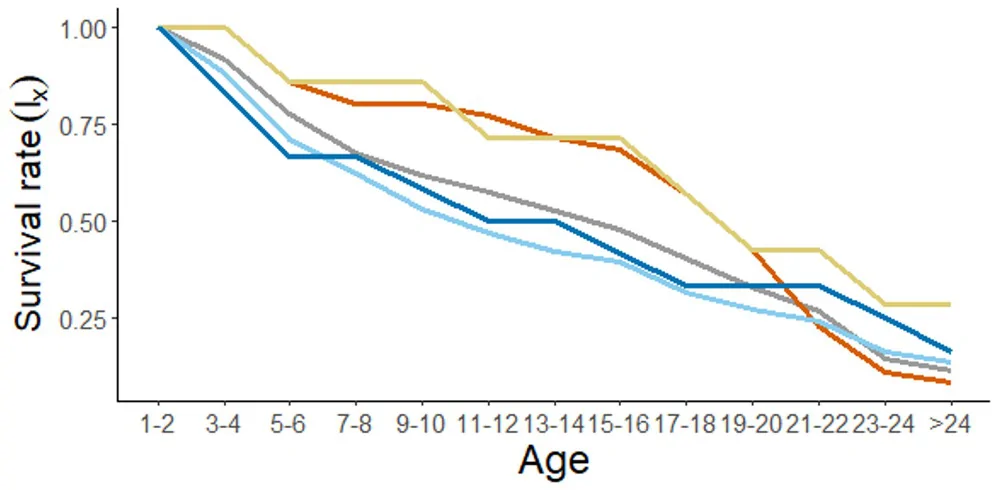
Survival by mitotype group for the two largest groups for each lineage. Total, Gray; J1A, Yellow; J1B, Orange; J2A, Light blue; J2B, Blue.

The two largest mitotype groups within each lineage did not differ in the age distribution of colony mortality (J1A vs. J1B: Kolmogorov–Smirnov Test, *D* = 0.29, *p* = 0.61; J2A vs. J2B: Kolmogorov–Smirnov Test, *D* = 0.12, *p* = 0.1). Within each lineage, the two largest mitotype groups of colonies did not differ in survival; for J1A and J1B (log‐rank test, *χ* ^2^ = 0, df = 1, *p* = 1), for J2A and J2B (log‐rank test, *χ* ^2^ = 0.7, df = 1, *p* = 0.4), and reflect the overall survival rate of the respective lineage (compare Figures 3B and 4).

### 3.7 Mother–Daughter Pairs

**FIGURE 5:**
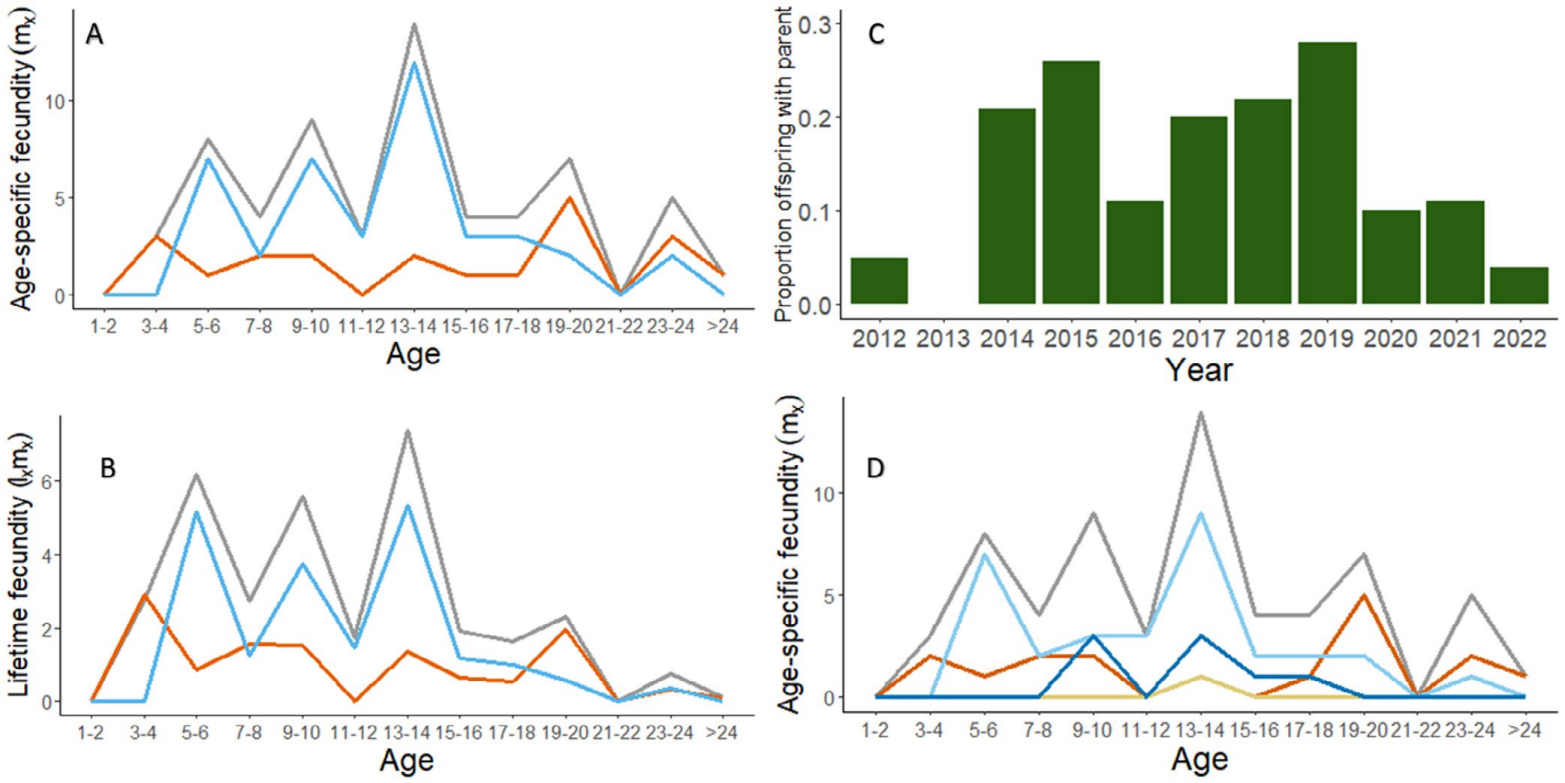
Fecundity for identified mother–daughter pairs of colonies, by lineage. (A) Age‐specific fecundity (m_x_) per age group by lineage. (B) Product of age‐specific survival and reproduction (*l*_*x*_ *m*_*x*_). Total, Gray; J1, Orange; J2, Blue. (C) Proportion of offspring colonies for which a parent colony was identified, by year D: Fecundity plot by mitotype group. Total, Gray; J1A, Orange; J1B, Yellow; J2A, Light blue; J2B, Blue.

Reduced‐representation genomic sequencing was used (ddRAD‐seq) to identify 62 mother–daughter colony pairs based on genetic relatedness: 21 pairs of J1 and 41 pairs of J2. The 62 pairs include 14 grandmother–mother–daughter triplets, consisting of 2 of these triplets from J1 and 12 from J2, including one great‐grandmother in J2. Overall, the peak in reproduction occurred at ages 11–17 years (*m*_*x*_ age‐specific fecundity, Figure 5A). The identified mother–daughter pairs of the two lineages differed in the distribution of age at reproduction (Kolmogorov–Smirnov test, *D* = 0.35, *p* = 0.02). In J1, the fecundity rate was low and steady, with a mean of 1.61 and a peak of 5 offspring in ages 19–20. In J2, the average was 3.15 offspring, with a peak of 12 offspring in ages 13–14 (Figure 5A). Colonies became grandmothers at ages 10–34. The number of offspring per parent by age group for J2 peaked at 5.33 for ages 13–14 and declined to 0 offspring per mother at ages older than 24 (*l*_*x*_ *m*_*x*_ as product of age‐specific survival and reproduction; Figure 5B).

From 2012 to 2022, the parent colony was identified for 28% of the offspring colonies (Figure 5C). This proportion differed among years, with a mean of 15% of colonies for which a parent colony was identified per year.

Mitotype groups within each lineage did not differ in the age distribution of mothers that were identified (Kolmogorov–Smirnov test, *D* = 0.25, *p* = 1). The distribution of mothers identified within mitotype groups was consistent with the overall numbers for each lineage (Table 5). The mothers identified in the two largest mitotype groups of J1 and J2 did not differ in age at reproduction (J1A and J1B, Kolmogorov–Smirnov Test, D = 0.5, *p* = 0.75; J2A and J2B, Kolmogorov–Smirnov Test, *D* = 0.29, *p* = 0.47). The mothers we identified in J1A and J1B show low, but constant fecundity, while J2A and J2B reached their maximum fecundity at the age of 11–17 and then decreased (Figure 5D). While the distribution of age at reproduction for mothers identified in the mitotype groups differs slightly from the overall distributions for each lineage (compare Figure 5A with Figure 5D), they show the same trends: similar fecundity across ages in J1 and a peak at ages 11–17 in J2. However, there were slight differences in fecundity of the mothers identified in J1A and J1B. In the 17 mothers we identified in J1A, fecundity declined after the age of 24; in J1B, we identified only two mothers, and they reproduced at ages 13 and 24.

## 4 Discussion

The lineage ratio was strongly skewed toward J2. The current ratio is consistent with the lineage ratio from other dependent‐lineage populations of *P. barbatus* and *P. rugosus* (Anderson et al. 2009; Gordon et al. 2013; Schwander et al. 2007). The proportion of the J2 lineage from 2012 to 2023 (mean (SD) 69.61 (4.67); Table 3) increased by about 10% from the ratio of 65.77 (mean (SD) 64.09 (2.05)) previously reported from 1986 to 2010 in the study population (Gordon et al. 2013).

The dependent lineage system introduces special challenges for the identification of kinship, because it creates genotype distributions in the hybrid workers that are very different from those of most sexually reproducing populations. The reproductive isolation of two lineages has led to divergent allele frequencies and the accumulation of fixed lineage‐specific alleles. The extreme and unusual distribution of heterozygosity (Figure S1) may distort many of the standard population genetic statistics and inference methods (e.g., Browning and Browning 2013; Lajmi et al. 2025), because it contradicts common assumptions underlying those methods, such as random mating. Here, we developed a novel approach for population genetic analysis based on the identification of lineage‐specific mitotypes from genomic sequencing data. Other studies of dependent‐lineage populations may be able to apply this approach.

We found no evidence for current intense selection on any heritable aspect of phenotype. Although we could identify only a small proportion of the mother–daughter pairs in the population, these were about equally distributed among mitotype groups. Since the mitotype is maternally inherited, all mother–daughter pairs must share the same mitotype. However, not all members of the same mitotype group are necessarily related, as there are only about 11 mitotypes (5 in J1, 6 in J2) in the population. If there were strong selection for a heritable phenotypic trait, we would expect to see differences among mitotype groups in recruitment or survival. Instead, each mitotype group of a lineage shows the fecundity characteristic of its lineage (Figure 5A,B vs. Figure 5D), and there were no significant differences in survival (Figure 4).

It appears that some families tend to dominate within each mitotype group. We identified 62 mother–daughter pairs of colonies, of which 14 (about 23%) are in grandmother–granddaughter triplets, and one great‐grandmother. Similarly, in a larger sample, Ingram et al. (2013) found 19 grandmother triplets (39% of all offspring), six great‐grandmothers, and one great‐great‐grandmother. Our results are consistent with the previous results showing that most reproduction occurs in only 25% of the potential parent colonies (Ingram et al. 2013).

We were able to identify some mother–daughter pairs, and the results show that the lineages differ in fecundity. This may contribute to the shift in lineage ratio toward J2. In the mother–daughter pairs we identified through 2023, fecundity was highest for mothers ages 11–17, mostly determined by reproduction by J2. Fecundity was higher in J2 colonies until the age of 13–14, while in J1 fecundity is constant up to age 20 and then drops (Figure 5A). This trend in fecundity differed from the results of a previous study through 2010 with a larger sample of mother–daughter pairs, which showed no trends toward reproductive senescence up to age 24 (Ingram et al. 2013). Ovarian state in *P. barbatus* changes with age, suggesting a decline in reproductive potential over time (Vergara‐Martínez et al. 2025). It is possible that this is more pronounced in J2 than in J1, leading to the drop in fecundity in J2 colonies after the age of 17. Despite this, J2 consistently produced more offspring than J1, increasing its proportion of the population. Overall, in both lineages, the number of new colonies per year has declined since drought conditions began in the early 2000s (Sundaram et al. 2022). In the mother–daughter pairs we identified from 2012 to 2023, the average number of offspring per mother colony was 1.23, with a range of 1–3 (Ingram et al. 2013). Before 2010, the average was 2.94, with a range of 1–8. In our sample, fecundity was highest for mothers ages 11–17, mostly determined by reproduction by J2.

The skewed lineage ratio raises the question of how the more common lineage, J2, can find enough mates of the rare lineage, J1. Just as sex ratios in animals are influenced by life history differences between the sexes (Kappeler et al. 2023), similar factors may help to maintain the two lineages. Previous work shows that the lineages are similar in number of patrilines per colony (means J1: 8.2, J2: 11) (Suni and Eldakar 2011), and each male mates only once (Gordon 2024). A *Pogonomyrmex* queen generally acquires sperm in the same ratio as the lineage ratio in the population (Helms Cahan and Gardner‐Morse 2013; Helms Cahan and Julian 2010; Helms Cahan et al. 2002; Helms Cahan and Vinson 2003).

There are several ways to maintain the population with a deeply skewed lineage ratio: either J1 colonies could produce more males so that each gyne on the mating aggregation can mate with a male from the rare lineage (Gordon et al. 2013), or J1 males could produce more sperm than J2 males. Workers may influence the sex ratio of reproductives in ant colonies (Boomsma and Grafen 1991; Chapuisat et al. 1997; Murakami et al. 2000; Sundström et al. 1996), so workers could act to increase the proportion of males.

Another possibility for maintaining the skewed lineage ratio involves the number of gynes produced by each lineage. Since the lineages are similar in number of patrilines (Suni and Eldakar 2011), the availability of mates of the rare lineage, J1, to gynes of J2 may be influenced by differences in the number or size of gynes produced by J2 colonies. In a study conducted in the 1990s, before the drought began, about 30% of the colonies in the population did not produce any reproductives. Older colonies tended to produce more reproductives but smaller females (Wagner and Gordon 1999), and it is possible that smaller females are less likely to survive (Keller and Passera 1989). As J2 colonies appear to show reproductive senescence, when the age distribution of J2 is skewed toward older colonies, this could diminish the availability of J2 gynes. The numbers of gynes could also be influenced by differences between the lineages in the consumption of reproductive female eggs (Helms Cahan et al. 2004; Schwander et al. 2006) or in the workers' care of reproductive and worker brood. Finally, female choice may allow the skewed sex ratio to persist. Because the two lineages differ in their cuticular hydrocarbon profile (Volny et al. 2006), gynes may be able to preferentially select males from the rarer lineage.

Our results suggest that ecological conditions may create differences between the lineages in allocation to reproduction and survival (Bell 1980; Lemaître and Gaillard 2017). Since the early 2000s, decreasing rainfall has led to a lower food supply for this seed‐eating species, and competition for foraging areas has increased (Sundaram et al. 2022). Even before 2000, only about 1% of female alates in the population managed to found viable new colonies (Gordon and Kulig 1998). Lineages may differ in the selection pressures on life history (Kirkwood and Rose 1991). We found differences between the lineages in mortality, fecundity, and age distribution during the period 2011–2023 when rainfall declined (Sundaram et al. 2022), suggesting there may be an effect of drought conditions on the survival of young colonies of J2 (Figure 3C). Colonies differ in the desiccation resistance provided by their cuticular hydrocarbons (Menzel et al. 2025); further work is needed to determine if there are differences between lineages in desiccation resistance.

Changes in the demography of this population since 1988 show that survival of new colonies is inhibited in years of low rainfall (Sundaram et al. 2022). Our study does not rule out the possibility of selection due to some heritable phenotypic trait, but it appears that the effects of intensifying drought conditions on survival may have more impact than the slower process of adaptation through natural selection.

Our results contribute to a growing awareness that global warming and changing precipitation may create new selection pressures on life history (Padda et al. 2021; Fischer et al. 2014) as well as on physiological response to water stress (Weaver et al. 2024). Small year‐to‐year differences in survival due to current conditions may have important demographic effects. For this desert ant species with a generation time of 5 years, it remains an open question whether selection can be rapid enough to make it possible to adapt to accelerating climate change.

## Author Contributions

**Felix Glinka:** formal analysis (equal), investigation (equal), methodology (equal), visualization (equal), writing – original draft (equal), writing – review and editing (equal). **Erik B. Steiner:** data curation (equal). **Eyal Privman:** conceptualization (supporting), funding acquisition (equal), methodology (equal), resources (equal), supervision (equal), writing – original draft (supporting). **Deborah M. Gordon:** conceptualization (lead), funding acquisition (lead), investigation (equal), methodology (equal), project administration (lead), resources (lead), supervision (lead), writing – original draft (lead), writing – review and editing (equal).

## Funding

This work was supported by the National Science Foundation (1940647) and United States ‐ Israel Binational Science Foundation (2019655).

## Conflicts of Interest

The authors declare no conflicts of interest.

## Supporting information


**Figure S1:** Count of loci with relative number of heterozygosity.
**Figure S2:** Number of colonies in the sample set found per year (A) and number of colonies in the sample set that died per year (B). J1 Orange, J2 Blue.
**Table S1:** Mitochondrial sequences of haplotype group by lineage.

## Data Availability

The scripts to find alleles belonging to one lineage and make a vcf homozygous for one lineage used in this study were submitted to the zenodo repository (https://doi.org/10.5281/zenodo.17459734). The ddRAD sequencing data are being processed to upload to NCBI (PRJNA1433433, PRJNA1433508, PRJNA1433511). The remaining data are deposited in the Stanford Data Repository (https://doi.org/10.25740/pj435tq4463).
