# Supplementary material for: Shifts in Demography in Changing Ecological Conditions in a Dependent‐Lineage Population of Harvester Ant Colonies
Source: Ecol Evol. 2026 Aug 23;16(8):e74228. doi: 10.1002/ece3.74228 (PMC13500799; doi:10.1002/ece3.74228)
Supplement: Supplementary file 1 [file ECE3-16-e74228-s001.docx]

## Supplementary

Supplementary Table 1: Mitochondrial sequences of haplotype group by lineage

| Haplotype Group | Mitochondrial sequence |
| --- | --- |
| J1 | |
| J1A | tttgactcctacctccatcaatctccttattaattttaagaaacttcattggaagaggtgttgggactggttgaactatttatccccctcttgcttcaaacatttttcacagaggtccttcaattgatatatctattttctcgttacatattgctggtatatcatcaattcttggagctattaattttattactacaattttaaatatacatcatataaactttactatagataaaatccctctcctagtatgatctatccttattacagctgtacttttattactatctctcccagtccttgctggagcaattactatactattaaccgatcgaaacttaaataccactttttttgatccaagtgggggaggggatcctattttatatcaacatttattttgattttttggtcatcccgaagtttatattttaatttacctgga |
| J1B | tttgactcctacctccatcaatctccttattaattttaagaaacttcattggaagaggtgttggaactggttgaactatttacccccctcttgcttcaaacatttttcacagaggtccttcaattgatatatctattttctcattacatattgctggtatatcatcaattcttggagctattaattttattactacaattttaaatatacatcatataaactttactatagataaaatccctctcttagtatgatctatccttattacagctgtacttttattactgtctctcccagtccttgctggggcaattactatactattaaccgatcgaaacttaaataccactttttttgatccaagtgggggaggggatcctattttatatcaacatttattttgattttttggtcatcccgaagtttatattttaattttacctgga |
| J1C | tttgactcctacctcctcaatctccttattaattttaagaaactttattggaagaggtgttgggactggttgaactatttatccccctcttgcttcaaacatttttcacagaggtccttcaattgatatatctattttctcgttacatattgctggtatatcatcaattcttggagctattaattttattactacaattttaaatatacatcatataaactttactatagataaaatccctctcctagtatgatctatccttattacagctgtacttttattactatctctcccagtccttgctggagcaattactatactattaaccgatcgaaacttaaataccactttttttgatccaagtggggggggggatcctattttatatcaacatttattttgattttttggtcatcccgaagtttatattttaatttttacctgga |
| J1D | tttgactcctacctcctcaatctccttattaattttaagaaacttcattggaagaggtgttgggactggttgaactatttatccccctcttgcttcaaacatttttcacagaggtccttcaattgatatatctatttctcgttacatattgctggtatatcatcaattcttggggctattaattttattactacaattttaaatatacatcatataaactttactatagataaaatccctctcctagtatgatctatccttattacagctgtacttttattactatctctcccagtccttgctggagcaattactatactattaaccgatcgaaacttaaataccactttttttgatccaagtgggggaggggatcctattttatatcaacatttattttgattttttggtcatcccgaagtttatattttaattacctgga |
| J1E | tttgactcctacctccatcaatctccttattaattttaagaaacttcattggaagaggtgttgggactggttgaactatttatccccctcttgcttcaaacatttttcacagaggtccttcaattgatatatctattttctcgttacatattgctggtatatcatcaattcttggagctattaattttattactacaattttaaatatacatcatataaactttactatagataaaatccctctcctagtatgatctatccttattacagctgtacttttattactatctctcccagtccttgctggggcaattactatactattaaccgatcgaaacttaaataccactttttttgatccaagtgggggaggggatcctattttatatcaacatttattttgattttttggtcatcccgaagtttatattttatttacctgga |
| J2 | |
| J2A | ttttactcctcccatctccttattaattctaagaaactttattggaggcggtgttgggactggatgaaccatttatcctcctcttgcttcaaatattttccatagaggtccctcagttgatatatctattttctcattacatattgctggtatatcatcaattcttggagctattaattttattactacaattttaaatatacatcacataaactttactatagataaaattcctctcttagtatgatctatcctcattacagcagtacttttattattgtctctcccagttctcgccggagcaattactatactattaactgatcgaaacttaaataccactttttttgatccaagaggagggggagaccctattttataccaacatttattttgattttttggccatcccgaagtttatattttatacctgga |
| J2B | tttgactcctccctccatcaatctccttattaattctaagaaactttattggaggcggtgttgggactggatgaaccatttatcctcctcttgcttcaaatattttccatagaggtccctcagttgatatatctattttctcattacatattgctggtatatcatcaattcttggagctattaattttattactacaattttaaatatacatcacataaactttactatagataaaattcctctcttagtatgatctatcctcattacagcagtacttttattattgtctctcccagttctcgccggagcaattactatactattaactgatcgaaacttaaataccactttttttgatccaaggggagggggagaccctattttataccaacatttattttgattttttggccatcccgaagtttatattttaattttacctgga |
| J2C | tttgactcctccctccatcaatctccttattaattctaagaaactttattggaggcggtgttgggactggatgaaccatttatcctcctcttgcttcaaatattttccatagaggtccctcagttgatatatctattttctcattacatattgctggtatatcatcaattcttggagctattaattttattactacaattttaaatatacatcacataaactttactatagataaaattcctctcttagtatgatctatcctcattacagcagtacttttattattgtctctcccagttctcgccggagcaattactatactattaactgatcgaaacttaaataccactttttttgatccagggggagggggagaccctattttataccaacatttattttgattttttggccatcccgaagtttatattttaattttacctgga |
| J2D | tttgactcctccctccatcaatctccttattaattctaagaaactttattggaggcggtgttgggactggatgaaccatttatcctcctcttgcttcaaatattttccatagaggtccctcagttgatatatctattttctcattacatattgctggtatatcatcaattcttggagctattaattttattactacaattttaaatatacatcacataaactttactatagataaaattcctctcttagtatgatctatcctcattacagcagtacttttattattgtctctcccagttctcgccggagcaattactatactattaactgatcgaaacttaaataccactttttttgatccaaggggaggaggagaccctattttataccaacatttattttgattttttggccatcccgaagtttatattttaattttacctgga |
| J2E | tttgactcctccctccatcaatctccttattaattctaagaaactttattggaggcggtgttgggactggatgaaccatttatcctcctcttgcttcaaatattttccatagaggtccctcagttgatatatctattttctcattacatattgctggtatatcatcaattcttggagctattaattttattactacaattttaaacatacatcacataaactttactatagataaaattcctctcttagtatgatctatcctcattacagcagtacttttattattgtctctcccagttctcgccggagcaattactatactattaactgatcgaaacttaaataccactttttttgatccaagaggagggggagaccctattttataccaacatttattttgattttttggccatcccgaagtttatattttaatttttacctgga |
| J2F | tttgactcctccctccatcaatctccttattaattctaagaaactttattggaggcggtgttgggactggatgaaccatttatcctcctcttgcttcaaatattttccatagaggtccctcagttgatatatctattttctcattacatattgctggtatatcatcaattcttggggctattaattttattactacaattttaaatatacatcacataaactttactatagataaaattcctctcttagtatgatctatcctcattacagcagtacttttattattgtctctcccagttctcgccggagcaattactatactattaactgatcgaaacttaaataccactttttttgatccagggggagggggagaccctattttataccaacatttattttgattttttggccatcccgaagtttatattttaatttttacctgga |

Supplementary Figure 1: Count of loci with relative number of heterozygosity.

Supplementary Figure 2: Number of colonies in the sample set found per year (A) and number of colonies in the sample set that died per year (B). J1 Orange, J2 Blue.
